# Supplementary material for: Examining sex differences in neurodevelopmental and psychiatric genetic risk in anxiety and depression
Source: PLoS One. 2021 Sep 2;16(9):e0248254. doi: 10.1371/journal.pone.0248254 (PMC8412369; doi:10.1371/journal.pone.0248254)
Supplement: Attachment — Submitted filename: Response to Reviewers.docx [file pone.0248254.s002.docx]

**Reviewer #1:**

**Given the higher prevalence of anxiety and depression in female, the authors took efforts to investigated sex differences of genetic liability in individuals with anxiety or depression using both PRS and CNVs. The two datasets (NCMH and PGC) the authors selected to perform exploration and replicatory analysis were appropriated dataset with sufficient phenotypic information and statistic power. The results were very clear with absence of robust sex effects on PRSs and rare CNV burden. Only weak evidence of higher ADHD PRS in females compared to males were detected, particularly in early onset group. The results provide a better understanding of sex differences of anxiety and depression adults were absent in genetic heterogeneity for both PRS and CNV burden.**

Many thanks for the positive comments and constructive feedback.

**Introduction.**

**Since the authors has been described the heritability of anxiety and depression using family/ twin study. The sex effects in those equation models have been summarized and also valuable to introduce to the readers, e.g., PMID 26649194, 16212676, 14531579.**

We have now added information to the introduction to indicate that the heritability estimates are similar for males and females.

**Methods.**

**PRS calculation: MHC region were excluded only when PRS-SCZ were calculated, why do not keep same protocol for other PRSs calculation? It is suggested to excluded more LD complex region (PMID: 18606306) to avoid PRS result bias.**

We have excluded the extended MHC region for calculating the schizophrenia PRS in line with typical practice in schizophrenia genetics research. As the reviewer clearly appreciates, this particular region shows both a very strong association signal for schizophrenia and high linkage disequilibrium. The latter means that the robust association signal in this region can be over-represented, which is generally dealt with by removal of the region from the analysis. For all other discovery phenotypes, there is no evidence that this region shows a strong association signal, therefore we applied the typical LD-clumping parameters (--clump-kb 500 --clump-r2 0.2), which will have limited the influence of variation in this region on the calculated PRS.

**PRSs were calculated for 1315 cases and 157 controls. The authors compared the PRSs only for cases between male and female after PRS standardized in total samples (including controls). As the different prevalence of cases in male compared in female, this standardization procedure may introduce bias for PRS sex comparison, which is suggested to excluded.**

Thank you for drawing this to our attention. In fact the PRS were re-standardised in the cases only for each analysis, but this was not clear. We have now clarified this in the Method section.

**Multiple testing correction. In Table S2, only PRS-ADHD, PRS-ANX and PRS-MDD showed significant association with case control status. I personally suggest only compare these 3 PRSs in sex groups in following study.**

In this analysis, we compared cases with any anxiety/depression to controls and found that PRS for ADHD, anxiety, MDD and ASD are higher in cases than controls. Although BD and schizophrenia PRS are not associated, the effects are in the expected direction, given previous evidence of shared genetic risks across these disorders. The small number of available controls means that we were under-powered to detect smaller effect sizes for this analysis. Despite no significant association with case-control status overall, it is still plausible that males and females with anxiety/depression could differ in polygenic burden for schizophrenia and bipolar disorder and therefore we performed an exploratory test of this.

**Still in Table S2, do PRSs contribute the variance of case-control status differently between male and female? It is good to show the summary statistics separately in male and female group also in table S2.**

We have now added these results separately for males and females (Table S2) and commented on them in the Results section.

**Genetic PCs, please explain the reason that authors select first 5 PCs out of 20 PCs as covariates into the models.**

In the NCMH sample, we found that the population ancestry variation captured by PCs beyond the top 5 PCs is minimal, reflecting the relatively small and ancestrally homogeneous nature of that sample. The same is true of the individual studies in the PGC MDD sample. We therefore included only these PCs in our analyses.

**Authors compared the sex difference of PRS and CNV burden separately. What if the comparison took both into account (e.g., Add CNV burden as covariates when comparing the PRSs)?**

In a multivariate analysis which includes both the presence of any large rare CNV and ADHD PRS, males and females do differ in terms of either CNV presence [OR(CIs)=0.78(0.51–1.19), p=0.25] or ADHD PRS [OR(CIs)=1.04(0.92–1.18), p=0.54]. These results are nearly identical to those presented in the univariate models in Tables 2 & 3, and do not contribute any further insights. This is to be expected since CNV burden and common variant PRS are independent of each other, and therefore including them together in a model does not impact the results.

**Results.**

**Table 2: the sex distribution is clear in total sample (63.2%); however, I could not find the sex distribution of early-onset samples and late-onset samples. Does sex distribution significant different from early-onset samples and late-onset samples? Please indicate this.**

We previously included information about the sex distribution of the early onset samples in NCMH in the final paragraph on page 12 (N=420, 74.8% female). We did not include information about the late-onset samples in NCMH (N=299, 58.5% female) as we do not analyse these samples separately, but in response to the reviewer, we have now included this information in the same paragraph on page 12.

**Discussion.**

**“It is plausible that a family history of psychiatric problems may motivate females to seek clinical help earlier and to be more likely to take part in mental health research, compared to males. Females might also be more aware of and better able to recall the mental health difficulties of their relatives.” This bias could be corrected by parenting report instead of self -report. To support this hypothesis, Chen et al found equal genetic component but larger shared environmental components in female compared with male in self-reporting data; and the equal shared, and nonshared environmental factors for both female and males using parent-report data.**

This is an interesting suggestion. Unfortunately we cannot address it as the majority of the NCMH sample (98.9%) are adults and parent-reports are not available.

**“Furthermore, family history of psychiatric disorders captures factors beyond genetic risk, including shared exposures and experiences (e.g., trauma or bereavement). We are unable to differentiate between these possibilities using our study design.” It would be great if authors consider other shared environmental factors given the possibilities of dataset; e.g., Smoking, alcohol comsuption and education attendance.**

Again, this is a good suggestion, but unfortunately we do not have data on shared familial environmental exposures in our dataset.

**Reviewer #2:**

**While there are a number of strengths to this study, there are a number of problems with the underlying premise and methods. Their premise is that women with depression and anxiety disorders will have greater genetic risk for neurodevelopmental disorders like ADHD and autism. This is surprising given that population and clinical studies have shown that people onsetting with major depression (clinically diagnosed) are less likely to have early cognitive deficits, deficits that are associated with neurodevelopmental disorders. In fact, boys/men have higher rates of ADHD, autism and other neurodevelopmental disorders. So, this reader is not sure from where their hypothesis emanated. Further, sex differences in depression emerge just post-puberty with young women onsetting at a greater rate than young men (and this holds across the lifespan as women have higher rates of depression than men). Finally, childhood onset depression does not show sex differences in incidence. (The age of <26 years as “early onset” does not reflect early onset.)**

We agree that neurodevelopmental disorders are more common in males than females, even in those individuals who have a diagnosis of depression, and indeed this is what we find in the NCMH sample. The rationale for our hypothesis boils down to the following. Previous studies show that autosomal genetic liability for neurodevelopmental disorder is approximately equally distributed in males and females. However, how this liability is manifested is not equal. Instead, males are more likely to be diagnosed with neurodevelopmental disorders than females, as the reviewer says. So we wanted to examine what is happening to the females with high liability to neurodevelopmental disorder. One hypothesis (which we test here) is that in females, this liability is manifest as depression/anxiety rather than as ADHD/neurodevelopmental disorder. If our hypothesis is true, then we predict that females with depression and anxiety (though not females in general) would be enriched for *genetic liability* to ADHD/neurodevelopmental disorders (not neurodevelopmental disorder *per se*) relative to males with depression and anxiety, with the males with high liability being diagnosed with neurodevelopmental disorder instead. This is supported by previous studies that we have cited, and we do find some limited evidence to support this hypothesis in the PGC MDD data, where we find that ADHD PRS are slightly increased in females compared to males with MDD.

We also agree that developmental differences are very important to consider. However, there were few childhood cases of depression available in both the NCMH and PGC datasets and there is evidence from other studies (e.g. Power et al, 2017) that depression prior to age 26 (which can be considered as “early onset” relative to the majority of depression cases) shows a different genetic risk profile to later onset depression. Therefore, we were only able to consider the timing of depression onset by using this broader “early onset” definition in our samples and we have acknowledged this as a limitation of our study.

**Perhaps the previous studies on this topic from which they drew their hypotheses are reflecting the fact that across all psychiatric disorders, women experience higher levels of depressive and anxiety symptoms, just like women in general in the population. This may be one reason why depressive/anxiety symptomatology is associated with many psychiatric disorders and by sex.**

**However, this is not telling us about major depression as a clinical diagnosis. It seems that the NCMH sample is more a reflection of symptomatology experienced rather than frank diagnoses. This is not a minor point - misclassification of disorder (based on self reports) can have different effects on findings than clinical diagnoses. The PGC data are a better reflection of the latter.**

We agree that self-report of having had a clinical diagnosis of anxiety or depression from a doctor or health professional is a limitation of the NCMH sample and discuss this in our limitations section. We also agree that this diagnostic difference between the PGC and the NCMH data may explain some of the differences in the results we observe in the analyses of the NCMH and PGC data. Nevertheless, there is evidence that even minimally phenotyped definitions of depression can be informative (e.g. Cai et al, 2020, PMID: 32231276), though results may not generalise to stricter phenotypic definitions and therefore need to be interpreted appropriately. We have now included this point in the Discussion (page 16).

**In addition, the men in their sample were significantly different clinicially - greater severity, higher rate of neurodevelopmental disorders, greater substance use disorders - characteristics we know reflect men with MDD. Again, not sure then from where the original hypothesis is emanating if men in the sample themselves have higher rates of neurodevelopmental diagnoses.**

**Are the authors suggesting that there may be higher genetic risk for neurodevelopmental disorders in women with depression that then are expressed as depression in women and not expressed in childhood as neurodevelopmental disorders? This seems convoluted.**

As outlined above, this indeed is what we are suggesting, though we do not agree that it is convoluted. It is well established that males are more likely to be diagnosed with neurodevelopmental disorders but there is also emerging evidence to suggest that ADHD and other neurodevelopmental disorders are likely under-diagnosed in females, particularly women with depression (see for example this recent pre-print: Powell et al, 2021; <https://www.medrxiv.org/content/10.1101/2021.02.05.21251211v1>). Shared genetic risks across psychiatric and neurodevelopmental conditions could manifest in a sex-specific manner and this may be an explanation of the sex differences in prevalence that are consistently observed. We think this is an important area for research.

**Additional point to address in the Introduction that stated no one had tested for sex differences in SNPS across disorders: Comorbidity of psychiatric disorders is pervasive and sex differences in shared SNPs were addressed for depression, bipolar disorder and schizophrenia in a large genome wide study in a recent paper in Biological Psychiatry by Blokland et al.**

We have now cited this recent paper (which was published after we submitted our manuscript) in our introduction, as follows: “The genetic correlation between males and females is very high for each of these disorders and there are no SNPs identified to date showing a genome-wide significant sex difference in allele frequency (12,13)”.
