# Supplementary material for: Examining sex differences in neurodevelopmental and psychiatric genetic risk in anxiety and depression
Source: PLoS One. 2021 Sep 2;16(9):e0248254. doi: 10.1371/journal.pone.0248254 (PMC8412369; doi:10.1371/journal.pone.0248254)
Supplement: Attachment — Submitted filename: Response to Reviewers.docx [file pone.0248254.s003.docx]

**Reviewer #1:**

**Although the authors mentioned that "the population ancestry variation captured by PCs beyond the top 5 PCs is minimal, reflecting the relatively small and ancestrally homogeneous nature of that sample.", the fraction of variance explained by top 5 PCs still not given in the manuscript, it is recommended to report. Thanks for the responses of the rest are clear.**

We have now added this information to the Supplemental Text, as follows:

“The cumulative variance explained by the top 5 PCs was 0.27% and no PC beyond that explained more than 0.0517% of the genotypic variance.”

**Reviewer #2:**

**Authors primarliy responded adequately to concerns.**

**One comment with respect to the diagnostic question of depression - The use of "self report of depressive symptomatology" as distinct from a clinical diagnosis of depression is not only a "minimally phenotyped definition" but may have genetic risk finding implications given that the latter is more likely to demonstrate genetic risk than the former. In fact, that may be one reason why the findings differ across samples. A comment as such is warranted or perhaps should be discussed with regard to implications for their findings.**

We have now added this point to our Discussion and clarified that this could explain our results:

“Although there is evidence that even minimally phenotyped definitions of depression can be informative (43), the results may not generalise to stricter phenotypic definitions. In particular, strict diagnosis may capture more severe and recurrent depression, which is more heritable (8), and such differences in phenotypic definitions could explain the different results observed in NCMH and PGC. The NCMH sample may be less severely affected compared to some of the clinically-ascertained samples included in the PGC MDD sample.”

**One additional minor note - In the introduction, the comment about “no SNPS showing genome-wide sex differences” gives the impression of a complete lack of sex differences in autosomal SNPs when this is not the complete story with regard to schizophrenia and major depression in the recent literature.**

We have now changed this statement as follows: “The genetic correlation between males and females is very high for each of these disorders and larger samples are needed to identify SNPs showing a genome-wide significant sex difference in allele frequency (12,13).”
